# Evaluation of the Mortality and Morbidity of Premature Infants During a Five-Year Period in the Neonatal Intensive Care Unit

**DOI:** 10.7759/cureus.17790

**Published:** 2021-09-07

**Authors:** Elif Çelik, Adnan Öztürk

**Affiliations:** 1 Department of Paediatrics, Adnan Menderes University, Aydın, TUR; 2 Pediatrics and Neonatology, Erciyes University, Kayseri, TUR

**Keywords:** respiratory distress syndrome, morbidity, mortality, maternal health, premature

## Abstract

Background

Premature birth is the most important cause of perinatal mortality and morbidity. This study aimed to evaluate mortality and morbidity in premature infants over a five-year period at a university hospital providing tertiary intensive care health services.

Methodology

All premature infants born alive at ≤37 gestational weeks and hospitalized in neonatal intensive care units were included in the study. Data such as maternal and perinatal characteristics, characteristics of the newborn, respiratory and related problems, neonatal morbidities, and causes of death were retrieved retrospectively from file records.

Results

A total of 1,780 patients (53.7% male and 46.3% female) were included in the study. High-risk pregnancy was present in 55% of women. Respiratory distress syndrome (RDS) developed in 50.4% of the patients, intracranial hemorrhage in 8.4%, and necrotizing enterocolitis in 5.6%. Mortality was observed in 20.9% of the patients. The most frequent cause of death was RDS and related complications (11.8%), and 66.4% of mortality occurred during the early neonatal period, that is, the first 24 hours of life.

Conclusions

High-risk pregnancies were significantly associated with neonatal morbidity and mortality. Therefore, the management of maternal health factors should be the priority for controlling neonatal mortality.

## Introduction

Premature birth is the most important cause of perinatal mortality and morbidity worldwide. Although prenatal care standards have improved and access to healthcare has increased in many countries, the incidence of premature birth continues to rise [1].

Although complete data on premature birth rates are not available in Turkey, it is estimated to be approximately 11.9% [2]. It is important to review neonatal statistics at specific intervals at the institutional or national level to determine the causes of death and underlying problems, to determine the success rate achieved with changes in care and therapeutic methods, and to adopt and develop effective preventive measures to reduce perinatal mortality. The present study aimed to evaluate mortality and morbidity in premature births at a tertiary university hospital.

## Materials and methods

The study was retrospectively conducted at a tertiary university hospital between January 1, 2003 and December 31, 2007. All premature infants born alive at ≤37 gestational weeks and hospitalized in neonatal intensive care units were included in the study. Patient data were obtained from file records.

Maternal and perinatal characteristics, maternal age, number of pregnancies, birth via in vitro fertilization (IVF) techniques, mode of delivery, cause of premature birth (such as preeclampsia, chorioamnionitis, premature rupture of membranes, and isolated preterm birth), characteristics of the newborn (such as gender and birth weight), respiratory and related problems, respiratory distress syndrome (RDS), surfactant treatment application, pulmonary hemorrhage, neonatal morbidities, presence of early and late sepsis, intracranial hemorrhage (ICH), hemodynamically significant patent ductus arteriosus (PDA) and congenital heart diseases, necrotizing enterocolitis (NEC), causes of mortality, and hospitalization periods as obtained from patient files were recorded in the case report forms prepared by the researchers. Furthermore, according to their birth weight, patients were divided into the following subgroups: 501-750 g, 751-1,000 g, 1,001-1,500 g, 1,501-2,000 g, 2,001-2,500 g, and >2,500 g [3].

Definitions

Infants with a gestational age of 37 weeks or less were considered premature [4]. RDS was diagnosed according to the clinical findings and chest radiography [5]. Surfactant was applied to the patients diagnosed with RDS according to their abnormal blood gas findings and chest radiography. The cause of death for the patients who died due to pneumothorax and pulmonary hemorrhage induced by RDS were included in the “RDS and related complications” group.

Early neonatal sepsis (postnatal less than seven days) and late neonatal sepsis (postnatal more than seven days) were diagnosed using blood culture growth [6,7]. ICH was diagnosed according to Papile classification via cranial ultrasound performed in the first 10 days and NEC was diagnosed according to modified Bell’s criteria [8-10].

Ethical issues

This study was approved by the Chief Physician of the hospital due to the retrospective design of the study and followed the principles for human investigations outlined in the Second Declaration of Helsinki.

Statistical analysis

Data were recorded and analyzed using SPSS version 16.0 (SPSS Inc., Chicago). Descriptive statistics were recorded as quantity and ratios and mean ± standard deviation (SD). The Chi-square test was used for statistical assessment, and p < 0.05 was considered to be significant.

## Results

The study included 1,780 patients (53.7% male and 46.3% female). Overall, 65.8% of the patients were born via cesarean section, and 34.2% were born via spontaneous vaginal delivery. The lowest gestational age was 22 weeks. The mean hospitalization period of the patients was 20.3 ± 22.9 days. The distribution of patients by gestational weeks is shown in Table 1. Overall, 39% of birth weights were between 1,501 g and 2,000 g (Table 2).

**Table 1:** Distribution of the gestational weeks of the study population.

Gestational week	22–23	24–25	26–27	28–29	30–31	32–33	34–37	Total
Number of patients (%)	16 (0.9)	51 (2.9)	102 (5.7)	203 (11.4)	312 (17.5)	462 (26.0)	634 (35.6)	1,780 (100)

**Table 2:** Distribution of the gestational weeks of the study population.

Birth weight (g)	501–750	751–1,000	1,001–1,500	1,501–2,000	2,001–2,500	>2,500	Total
Number of patients (%)	63 (3.5)	161 (9.1)	501 (28.1)	694 (39)	291 (16.4)	70 (3.9)	1,780 (100)

The mean maternal age was 26.2 ± 5.8 years, ranging between 15 and 50 years. The analysis showed that 75.1% of the neonates were delivered in the hospital’s tertiary gynecology-obstetrics unit. The antenatal steroid use rate was 28.1%. In total, 55% of the mothers had a history of high-risk pregnancy. Eclampsia/preeclampsia was present in 12.4% of the mothers, diabetes mellitus in 6.2%, urinary tract infection in 4.2%, and chorioamnionitis in 8.4% (Table 3). The mean length of hospitalization among the patients was 20.3 ± 22.9 days.

**Table 3:** Maternal characteristics of the study participants.

Variables	Number	Percentage
Maternal age (years)		
<18	186	12.3
19–34	1,096	72.1
35+	237	15.6
Gravidity		
<2 pregnancies	450	29.6
≥2 pregnancies	1,069	70.4
Place of delivery		
Gynecology-Obstetrics	1,155	75.1
Other health facilities	213	14.7
Home delivery	151	10.2
Maternal status		
Healthy	684	45
Eclampsia/Pre-eclampsia	188	12.4
Diabetes mellitus	94	6.2
Urinary tract infection	62	4.2
Chorioamnionitis	129	8.4
Other	362	23.8
Antenatal visit		
Yes	1,190	78.4
No	329	21.6
Mode of delivery		
Spontaneous vaginal delivery	519	34.2
Cesarean section	1,000	65.8
Antenatal steroid		
Yes	426	28.1
No	1,093	71.9

The incidence of RDS by birth weight is shown in Table 4. The total incidence of RDS was 50.4%. For infants weighing <1,001 g, 1,001-1,500 g, 1,501-2,000 g, 2,001-2,500 g, and >2,500 the incidence of RDS was 77.2%, 56.3%, 37.7%, 48.1%, and 58.6%, respectively.

**Table 4:** Prevalence of respiratory distress syndrome according to birth weight.

Birth weight (g)	Respiratory distress syndrome		Total
	No [n (%)]	Yes [n (%)]	
501–750	14 (22.2)	49 (77.8)	63
751–1,000	37 (23)	124 (77)	161
1,001–1,500	219 (43.7)	282 (56.3)	501
1,501–2,000	432 (62.3)	262 (37.7)	694
2,001–2,500	151 (51.9)	140 (48.1)	291
>2,500	29 (41.4)	41 (58.6)	70
Total	882 (49.6)	898 (50.4)	1,780

The total incidence of RDS was 50.4%, with rates of 94%, 60.4%, and 33% reported for babies born at <28 weeks, 28-32 weeks, and 32-37 weeks, respectively (Table 5). RDS was most frequently seen in infants with birth weights ranging between 501 g and 750 g and those born under 28 weeks. Surfactant therapy was applied to 30% of the patients with RDS.

**Table 5:** Incidence of respiratory distress syndrome by gestational age. RDS: respiratory distress syndrome

	<28 weeks [n = 169 (9.9%)]	28–32 weeks [n = 746 (41.9 %)]	32–37 weeks [n = 865 (48.6 %)]	Total (n = 1,780)
RDS	159 (94%)	451 (60.4%)	288 (33%)	898 (50.4%)

Overall, 20.9% of the patients died. There was no significant difference between genders in terms of mortality rates (p > 0.05). No cause of death other than prematurity was determined in 372 patients (11%). The most frequent cause of death was RDS and related complications (11.8%). The mortality rate in the RDS patient group with birth weights of 501-750 g was significantly higher than those with other birth weights (p < 0.05). The mortality rate associated with RDS decreased as the birth weight increased (Table 6).

**Table 6:** Distribution of mortality rates in patients with respiratory distress syndrome according to birth weight.

Birth weight (g)		Mortality		Total
		No	Yes	
501–750 (n = 63)	n	1	48	49
	%	2.1	97.9	
751–1,000 (n = 161)	n	29	95	124
	%	23.4	76.6	
1,001–1,500 (n = 501)	n	181	101	282
	%	64.2	35.8	
1,501–2,000 (n = 694)	n	240	22	262
	%	91.6	8.4	
2,001–2,500 (n = 291)	n	118	22	140
	%	84.3	15.7	
>2,500 (n = 70)	n	38	3	41
	%	92.7	7.3	
Total (n = 1,780)	n	607	291	898
	%	67.6	32.4	

In addition, there was a statistically significant decrease in the mortality rates caused by ICH and NEC with an increase in birth weight (p < 0.05). Table 7 lists the causes of death and related complications.

**Table 7:** Causes of mortality determined in the patients. RDS: respiratory distress syndrome; ICH: intracranial hemorrhage; DIC: disseminated intravascular coagulation; NEC: necrotizing enterocolitis; CHD: congenital heart disease; IP: intestinal perforation

Cause of death	Number of patients		Cause of death	Number of patients		Cause of death	Number of patients	
	n	%		n	%		n	%
RDS	44	11.8	ICH, DIC	2	0.5	RDS, pneumonia, ICH	1	0.3
Pneumothorax	43	11.5	Pneumonia, DIC	2	0.5	RDS, pneumonia, DIC	1	0.3
Prematurity	41	11.0	Pneumothorax, ICH	2	0.5	RDS, pneumonia, DIC, NEC	1	0.3
Pneumonia, RDS	34	9.1	DIC, NEC	2	0.5			
RDS, pneumothorax	26	6.9	RDS, ICH, DIC	2	0.5			
Pneumonia	18	4.8	Pneumonia, pneumothorax, DIC	2	0.5			
Sepsis	17	4.6	Pneumonia, pneumothorax, DIC, sepsis	2	0.5			
DIC	15	4.0	Pneumothorax, NEC	1	0.3			
ICH	14	3.7	IP	1	0.3			
Pneumonia, pneumothorax	11	2.9	RDS, NEC	1	0.3			
CHD	10	2.7	Pneumonia, DIC, CHD	1	0.3			
DIC, sepsis	10	2.7	Pneumothorax, DIC, sepsis	1	0.3			
RDS, pneumonia, pneumothorax	9	2.4	Pneumothorax, DIC, CHD	1	0.3			
NEC	6	1.6	DIC, sepsis, CHD	1	0.3			
RDS, ICH	5	1.3	RDS, pneumonia, pneumothorax, NEC	1	0.3			
RDS, sepsis	4		RDS, pneumonia, ICH, DIC	1	0.3			
Pneumothorax, sepsis	4	1.0	DIC, CHD	1	0.3			
Sepsis, NEC	4	1.0	DIC, IP	1	0.3			
DIC, sepsis, NEC	4	1.0	Pneumonia, pneumothorax, DIC, CHD	1	0.3			
RDS, DIC	3	0.8	Sepsis, IP	1	0.3			
ICH, sepsis	3	0.8	NEC, CHD	1	0.3			
Pneumothorax, DIC	3	0.8	Pneumothorax, ICH, sepsis, NEC	1	0.3			

In terms of mortality, 23.4% of the deaths were reported in the first 24 hours of life, 66.4% in the early neonatal period including the first 24 hours, 23.4% in the late neonatal period, and 10.2% in the postneonatal period. The highest mortality rate in the early neonatal period was in the group with birth weights of 501-750 g (78.5%). Table 8 lists the times of death by the birth weights of the infants.

**Table 8:** Distribution of times of death according to birth weights.

Birth weight (g)		0–24 hours	2–7 days	8–28 days	More than 29 days	Total
501–750	n (%)	16 (28.5)	28 (50)	10 (17.8)	2 (3.7)	56 (100)
751–1,000	n (%)	21 (20.1)	51 (49)	24 (23.1)	8 (7.8)	104 (100)
1,001–1,500	n (%)	27 (20.9)	48 (37.2)	37 (28.79	17 (13.2)	129 (100)
1,501–2,000	n (%)	9 2 (2.5)	14 (35)	8 (20)	9 (22.5)	40 (100)
2,001–2,500	n (%)	11 (33.3)	15 (45.4)	6 (18.2)	1 (3.1)	33 (100)
>2,500	n (%)	3 (30)	4 (40)	2 (20)	1 (10)	10 (100)
Total	n (%)	87 (23.4)	160 (43)	87 (23.4)	38 (10.2)	372 (100)

Overall, 7.5% of the patients were born via IVF. In total, 23.3% of the infants born via spontaneous pregnancy were born from multiple pregnancies. The rate of multiple pregnancies was 92.5% among infants born via IVF, and the difference was statistically significant (p < 0.05). Echocardiography was performed for 18.8% of the patients, and PDA was detected in 17.6% of the patients.

## Discussion

Despite increasing innovation and technological advancement in the fields of neonatology and perinatology, premature birth rates are still rising. Approximately 80% of preterm deliveries are reported to occur spontaneously as preterm labor (50%) or preterm rupture of membranes (30%). The remaining 20% of preterm deliveries involve interventions for maternal or fetal problems including antenatal care follow-up [11], postnatal care follow-up [11], and complications during pregnancy [12,13]. Consequently, severe complications such as RDS, ICH, PDA, NEC, and sepsis may occur [14].

RDS is an important concern among preterm infants and is a significant cause of neonatal mortality. While Jaberi et al. reported that RDS occurred in 23.8% of all premature neonates, Caner et al. showed a 40.6% incidence of RDS among premature neonates [15,16]. The Vermont Oxford Network (VON) 2008 data indicated an incidence in RDS of 30%, specifically RDS incidence of 91%, 64%, 35%, 24%, and 16% for infants weighing <1,001 g, 1,001- 1,500 g, 1,501-2,000 g, 2,001-2,500 g, and >2,500 g, respectively [17]. The total incidence of RDS in the present study was 50.4%, with rates of 77.2%, 56.3%, 37.7%, 48.1%, and 58.6% in the different birth weight groups. The incidence of RDS according to the birth weight determined in the present study is higher than that reported in developing countries. However, the incidence of RDS according to gestational age is similar to that reported in developing countries. Therefore, we conclude that higher RDS rates in this study can be attributed to the greater number of patients with lower birth weeks in our cohort.

An RDS incidence of 58.6% was reported for babies with a birth weight of >2,500 g in the present study, whereas the VON 2008 data indicated an incidence of 30% [17]. High-risk pregnancy was present in 55% of the pregnant women in the present study, which is higher than that reported by numerous studies [12,18,19]. In addition, the antenatal visit rate was lower than that reported in previous studies [12,18-20].

Antenatal steroid intake reduces the risk of RDS [14]. However, based on the National Institute of Child Health and Human Development data, the antenatal steroid intake ranges between 47% and 90% [21]. In our view, the high rate of RDS in the present study can be attributed to the low rate of antenatal steroid (25.2%) administration in our hospital. In addition, because our hospital is the only center providing tertiary neonatal intensive care services in the region, it is also a reference center for high-risk pregnancies, which might have resulted in a higher incidence of RDS.

In this study, 51.4% of the premature infants born before the 32nd gestational week required surfactant therapy. Similarly, Heljić et al. showed that approximately 50% of premature infants born before the 30th gestational week require surfactant therapy [22]. Dani et al. applied surfactant therapy to 45% of 562 infants with RDS [23]. Caner et al. reported a rate of surfactant administration of 38%, with 14.6% of all patients being born at ≤28 weeks. Consistent with Caner et al., surfactant therapy was administered to 30% of the patients diagnosed with RDS in the present study [16]. However, only 9.9% of our patients were born under 28 weeks, which may also be due to the lower rate of surfactant therapy.

ICH is a major complication linked to long-term neurological sequelae in preterm infants. Although the incidence of ICH has decreased following improvements in neonatal intensive care in recent years, it remains an important concern in preterm infants with very low birth weight. Handley et al. reported an incidence of ICH of 15% in preterm infants born at less than 32 gestational weeks [24]. In a study from Turkey, Ozdemir et al. reported that the ICH incidence was 32% [25]. While the incidence of ICH was 45% before 1980, it decreased to 12-30% in the 1990s. The VON 2008 data reported an incidence of ICH ranging between 5.6% and 12.5% [17]. The ICH incidence in the present study was 8.4%, which is consistent with recent literature.

Together with an increase in the diagnosis of PDA in recent years, the rate of medical therapy in PDA decreased from 32% to 18% [26]. Emiroglu et al. reported a diagnosis rate of 21.9% for PDA [27]. The rate of diagnosis for PDA in the present study was 17.6%, which can be attributed to the higher gestational age of our patients and fewer cardiology visits in the neonatal unit.

NEC is the most important gastrointestinal emergency in premature infants. Caner et al. determined a 6.6% incidence of NEC in infants younger than 37 gestational weeks [16]. Horbar et al. reported an incidence of NEC between 3% and 9% [17]. Tayman et al reported an incidence of NEC of 11.4% among preterm infants younger than 32 gestational weeks [28]. Consistent with previous research, the incidence of NEC in this study was 5.6%.

Multiple pregnancies via assisted reproductive techniques increase the incidence of preterm births. A previous study showed that the risk of premature birth increased by 16.2% among singletons, 55% among twins, and 76% among triplets, indicating that pregnancy status among infants born via assisted reproductive techniques may be a risk factor for premature birth [29]. Similarly, the proportion of infants born via assisted reproductive techniques in this study was 7.5%, 92.5% of whom were from multiple pregnancies. A study evaluating all premature infants irrespective of birth weight reported a mortality rate of 29.7% [30] compared to 14.1% reported by Caner et al. [16]. The mortality rate in the present study was 20.9%.

There are several limitations to the present study. Because the patient data were collected retrospectively, no information regarding the number of antenatal steroid administration was available. In addition, maternal socioeconomic status, another crucial risk factor for neonatal mortality, was not investigated. A common risk factor of neonatal mortality, such as mothers, was also not analyzed.

## Conclusions

High-risk pregnancies are significantly associated with neonatal morbidity and mortality. Therefore, the management of maternal health factors represents a priority for controlling neonatal mortality.
